# Exclusive breastfeeding and associated factors among HIV positive mothers in Northern Ethiopia

**DOI:** 10.1371/journal.pone.0210782

**Published:** 2019-01-16

**Authors:** Negeso Gebeyehu Gejo, Haftom Gebrehiwot Weldearegay, Kidisti Tesfay W/tinsaie, Dejene Ermias Mekango, Ermias Sahile Woldemichael, Alula Seyum Buda, Leta Hinkosa Dinsa, Mussie Alemayehu, Gelila Goba

**Affiliations:** 1 Department of Midwifery, College of Medicine and Health Sciences, Wachemo University, Hosanna, Ethiopia; 2 Department of Midwifery, College of Health Sciences, Mekelle University, Mekelle, Ethiopia; 3 Department of Midwifery, College of Health Sciences, Mekelle University, Mekelle, Ethiopia; 4 Department of Public Health, College of Medicine and Health Sciences, Wachemo University Hosanna, Ethiopia; 5 Department of Midwifery, College of Medicine and Health Sciences, Mizan-Tepi University, Mizan, Ethiopia; 6 Department of Nursing, College of Medicine and Health Sciences, Wachemo University, Hosanna, Ethiopia; 7 Department of Midwifery, College of Medicine and Health Sciences, Wollega University, Nekemte Ethiopia; 8 Department of Public Health, College of Health Sciences, Mekelle University, Mekelle, Ethiopia; 9 Department of Obstetrics and Gynecology, University of Illinois at Chicago, Chicago, Illinois; University 9 of July, BRAZIL

## Abstract

**Background:**

It is estimated that sub-optimal feeding, especially non-exclusive breastfeeding in the first 6months of life, results in 1.4million deaths and 10% of disease burden in children younger than five years. Worldwide, it is estimated that only 34.8% of infants are exclusively breastfed for the first 6months of life, the majority receiving some other food or fluid in the early months. Besides, the Ethiopian demographic and health survey (2016) stated that the median duration of exclusive breastfeeding in Tigray region was 3.8 months which is shorter than the recommended duration. The main purpose of this study was to determine the magnitude of exclusive breastfeeding practice and associated factors among HIV positive mothers in public hospitals of Tigray region, Northern Ethiopia.

**Methods:**

A facility based cross-sectional study was conducted from July 9 to October 11, 2016, in public hospitals of Tigray region. Data was collected by using structured questionnaire using face-to-face interview among 304 eligible women through a systematic sampling technique. Data was analyzed using SPSS version 20.0. Binary and multiple variable logistic regressions (“odds ratio”) analyses were calculated with 95% CI and p value ≤ 0.05 as significance were used.

**Result:**

Two hundred seventy (88.8%) of mothers practiced exclusive breastfeeding for the first six months of life. Infant feeding counseling during antenatal care of last pregnancy [AOR = 6.9, 95% CI; 2.63, 17.99], knowledge on exclusive breastfeeding (AOR = 5.5, 95% CI; (2.12, 14.02] and attitude towards exclusive breastfeeding [AOR = 7.9; 95% CI; 2.96, 21.21] had significant association with exclusive breastfeeding practice.

**Conclusions:**

A high proportion of mothers practiced exclusive breastfeeding for the first six months of life. Infant feeding counseling, knowledge and attitude towards exclusive breastfeeding practice were the predictors of exclusive breastfeeding among HIV positive mothers. Strengthening infant feeding counseling during antenatal care and improving mothers’ knowledge and attitude on exclusive breastfeeding is essential.

## Background

Exclusive breastfeeding (EBF) is defined as feeding the infant only breast milk, and no additional liquids or solids, not even water, apart from drops or syrups consisting of vitamins, minerals supplements or medicines[1]. Exclusive breastfeeding in the first 6 months of life boost babies’ immune system and protects them from diarrhea and acute respiratory infections which are leading causes of infant mortality in the developing world[2].

Globally, 36.9 million [31.1 million-43.9 million] people were living with human immunodeficiency virus (HIV) in 2017. 1.8 million [1.4 million-2.4 million] people became newly infected with HIV in 2017. 940 000 [670 000–1.3 million] people died from acquired immunodeficiency syndrome (AIDS) related illnesses in 2017[3].

The risk of HIV transmission through breast milk and preserving its life saving benefit is challenge for many researchers, policy makers and HIV positive mothers particularly those from developing countries[4].

Worldwide, breastfeeding bring about 300,000 HIV infections per year while at the same time, United Nations Children’s Emergency Fund (UNICEF) estimated that not breastfeeding is responsible for 1.5 million children death per year. The largest burden of these infection and deaths occur in sub-Saharan Africa. Studies revealed that exclusive breastfeeding for the first six months of life after birth reduces the risk of postpartum transmission of HIV from an infected mother to her baby[5, 6].

The overall prevalence of exclusive breastfeeding in sub-Saharan Africa (SSA) was low which was 36%, the prevalence was highest in Rwanda and lowest in Gabon, despite the fact that sub-Saharan Africa (SSA) is the home for higher rates of mother to child HIV transmission (MTCT), malnutrition, infant and child mortality rates[7].

According to Ethiopian demographic and health survey (EDHS) 2016 report, 58% of children under age 6 months are exclusively breastfed, and the percentage of exclusive breastfeeding declines with age from 74% in 0–1 months to 36% in 4–5 months. Besides, EDHS 2016 report stated that the median duration of exclusive breastfeeding in Tigray region was 3.8 months[8].

Several studies were conducted on infant nutrition practice in Ethiopia. However, there is still a gap in the assessment of HIV positive mothers in relation to their practice towards exclusive breastfeeding and associated factors. Therefore, this study aims to investigate factors associated with exclusive breastfeeding practice among HIV positive mothers in the public hospitals of Tigray region, Northern Ethiopia, so that it provides evidence based information for improving and enhancing the practice.

## Methods and materials

A facility based cross-sectional study design was conducted from July 9 to October 11, 2016 in four randomly selected public hospitals of Tigray region. Tigray regional state is located at the northern part of Ethiopia. It has an estimated area of 54,569.25 square kilometers. The region is divided into 7 zones and 46 districts; out of which 34 are rural and 12 are urban. Mekelle is the capital city of the region.

The source populations were all HIV positive mothers of infants aged 6–12 months, who attended antiretroviral therapy (ART) and prevention of mother-to-child transmission of HIV (PMTCT) clinics in public hospitals of Tigray region. The study population is composed of HIV-positive mothers of infants aged 6–12 months, who visited one of the selected public hospitals for ART and prevention of mother-to-child transmission of HIV (PMTCT) during data collection period.

The appropriate sample size was determined using a single population proportion formula by considering the following assumptions; 95% confidence level, 5% margin of error, and proportion of HIV positive mothers who practiced recommended infant feeding options (75.4%) [9]. Adjusting for 5% non- response, the minimum required sample size was determined to be 304.

There are 15 governmental general hospitals in the region. First, we listed all 15 governmental general hospitals. After this, 4 governmental general hospitals were selected by using lottery method (simple random sampling technique). In the selected hospitals, the sampling frame of the HIV exposed infants (HEIs) attending PMCT/ART service who were 6–12 months (during the study period) was obtained from registration book of each health facilities. Probability proportional to size (PPS) was used to determine the appropriate sample size for the respective hospitals. Finally, a systematic sampling technique was employed to determine the sampling method of study participants. The recruitment interval was calculated to be 3 and the starting point was found to be the 3rd mother after using lottery method.

The questionnaire was first prepared in English, translated to Tigrigna, and then translated back to English to check for consistency. It was checked for its consistency by translating it back to English by two different individuals. Data was collected by Tigrigna, which is the local language. Data was collected by pretested and structured questionnaire using face to face interview. Data was collected by 4 midwives with diploma qualification. Training was given to data collectors and supervisors. The training was given on general aspect structured questionnaire, problem and objectives of the study and how to record responses. The interview was conducted during exit period after mothers got services and has three stages which are 'Introduction and rapport building'; 'Interviewing' and 'Closing the interview'. The interview was conducted in a place where the woman feels free to express her feelings and ideas. The questionnaire was adapted from related published literature by considering local situation of study area and objective[10, 11]. The validity of instrument was assured by evaluating three criteria’s. These are 'homogeneity' (the instrument only measured one construct), 'convergence' (the instrument was poorly correlated to the instruments that measure different variables) and 'predictive value' (the instrument had high correlations with future criterions). Reliability was assured by stability (the instrument was given to the same participants more than once under similar circumstances and it was consistent).

The questionnaire was pre-tested on 5% (15) of the calculated sample size in a hospital which was not included in the study (Adwa hospital) preceding the actual data collection period. The questionnaire encompassed six parts; socio-demographic information, disclosure of HIV status, infant feeding practice, knowledge of mothers on EBF practice, attitude of mothers towards EBF practice and obstetrics variables. The data collection process was strictly supervised and data checked for consistency and completeness.

Eligibility criterion was all HIV positive mothers having infants aged 6–12 months visiting selected public hospitals of Tigray region for ART and PMTCT during the study period. Those mothers and/or child who were critically ill excluded from the study.

### Measurement

*Knowledge about EBF* was measured by using ten questions. Those respondents who score ≥6 was taken to 'good knowledge' and <6 taken as 'poor knowledge' [12].

*Attitude towards EBF* was measured using 5 points Likert scale. There were a total of 12 questions assessing attitude of mothers. The order of scoring for positive statements was (strongly agree = 5, agree = 4, undecided = 3, strongly disagree = 2, disagree = 1) and vice versa for negative statement statements. The total score was obtained and computed for mean so as to categorize it in to favorable and unfavorable attitudes. Score ≥ mean was taken as ‘favorable attitude’, while score < mean was taken as ‘unfavorable attitude’[13].

### Data analysis

Data was entered, cleaned and analyzed using SPSS 20. Tables with frequency and percentage (%) were used to represent results of categorical variables like age, income, religion, ethnicity, occupation status, educational level, residence, marital status, disclosure of HIV status, knowledge and attitude of HIV positive mothers, ante natal care, infant feeding counseling, mode of delivery, place of delivery and time at initial breastfeeding.

Both bivariate and multivariable logistic regression analyses were used to determine the association of independent variables with the dependent variable. Variables with p<0.2 in bivariate analysis were entered into a multivariable logistic regression model to adjust the effects of cofounders on the outcome variable. Odds ratios with 95% confidence interval were computed to identify the presence and strength of associations, and statistical significance was declared if p < 0.05 was found. Values will be displayed in COR and AOR. The Hosmer-Lemeshow statistic had chi-square value of 5.113 and a significance of 0.745 which means that Hosmer-Lemeshow test is not statistically significant and therefore the model is quite a good fit. Multi-collinearity were checked for interaction by VIF (Variance inflation factor) which is less than 5.

### Ethics approval and consent to participate

The study protocol was approved by the Institutional Research Review Board of Mekelle University’s College of Health Sciences and Community Services Ethical Review Committee (reference number ERC 07878/2016). Permission was obtained from all relevant authorities in the Tigray Regional Health Bureau and hospitals. Informed written consent was obtained from participants prior to enrollment in the study. For participants who were unable to write, a right thumbprint was taken as a signature. Parental or legal guardian consent was obtained for respondents who were under 18 years of age. Participation in the study was voluntary and participants were informed of the right to withdraw from the study. Data collection was conducted confidentially and data de-identified and de-linked and stored in a secure location.

## Result

### Socio-demographic characteristics of study participants

A total of 304 study participants were interviewed, with a response rate of 100%. Of these respondents, 223 (73.4%) were aged between 25–34 and 42 (13.5%) were aged 35 and above. 281 (92.4%) were from urban, 289 (95.1%) were Tigre, 284 (93.4%) were Orthodox and 250 (88.2%) were married. 115 (37.8%) had followed secondary education and 84 (27.6%) had no formal education.

With regard to their occupation, nearly half of the participants 146 (48%) were housewives and 15 (4.9%) were farmers. 117 (38.5%) and 115 (37.8) of respondents have 2 and 3–5 children respectively. More than half of the respondents 175 (57.6%) earn a monthly income of 1001 birr and above (> = 45.55 USD). About 187 (61.5%), 117 (38.5%), 167 (54.9%) infants were aged between 6–8 months, 9–12 months and female respectively (Table 1).

**Table 1:** Socio-demographic characteristics of HIV positive women visiting public hospitals of Tigray region from July to October 2016 (N = 304).

**Variables**	Category	Frequency	Percentage
Age	15–24	39	12.8
	25–34	223	73.4
	> = 35	42	13.8
Marital status	Married	250	82.2
	Single	27	8.9
	Divorced	24	7.9
	Widowed	3	1.0
Ethnicity	Tigre	289	95.1
	Amhara	15	4.9
Religion	Orthodox	284	93.4
	Muslim	16	5.3
	Catholic	4	1.3
Educational status	No formal education	84	27.6
	Primary education	83	27.3
	Secondary education	115	37.8
	More than secondary education	22	7.2
Place of residence	Urban	281	92.4
	Rural	23	7.6
Occupation	Government employee	57	18.8
	Merchant	54	17.8
	Farmer	15	4.9
	House wife	146	48.0
	Daily laborer	32	10.5
Monthly income	<22.75 USD*	19	6.3
	22.78–45.5 USD	110	36.2
	> = 45.55 USD	175	57.6
Age of infant	6–8 months	187	61.5
	9–12 months	117	38.5
Sex of infant	Male	137	45.1
	Female	167	54.9

*1ETB = 0.00455USD

### Previous obstetrics characteristics and HIV disclosure status

Among 304 respondents, 281 (92.4%) disclosed their HIV status to their spouse. A majority of mothers (66.7%) from those who did not disclose their status did so because of fear of divorce.

Two hundred seventy-nine (91.8%) attended antenatal care (ANC) service during their last pregnancy and 257 (84.5%) mothers had received infant feeding counseling during the ANC service.

Two hundred ninety (95.4%) respondents gave birth between thirty-seven and forty-two weeks of gestational age (term) and all mothers 304 (100.0%) delivered at health facility. From the total respondents, 268 (88.2%), 28 (9.2%) and 8 (2.6%) had spontaneous vaginal delivery, instrumental delivery, and Cesarean section respectively. Only 20 (6.6%) of women experienced breast problem. Of which less production of milk (75.0%) was the most common one followed by breast engorgement (15.0%).

### Knowledge and attitude towards exclusive breastfeeding practice

Two hundred forty three (79.9%) knew that HIV virus transmits from mother to child during pregnancy, delivery and breastfeeding and 3 (1%) didn’t know when HIV virus transmits from mother to child (Table 2).

**Table 2:** Knowledge of when MTCT of HIV occurs of HIV positive women visiting public hospitals of Tigray region from July to October 2016(N = 304).

**When HIV virus does transmit from the mother to child? (N = 304)**	Frequency	Percentage
During pregnancy only	31	10.2
During delivery only	6	2.0
During breastfeeding only	21	6.9
During pregnancy, delivery and breastfeeding	243	79.9
I don’t know	3	1.0
Total	304	100.0

Two hundred sixty (85.5%) respondents had good knowledge concerning exclusive breastfeeding practice. 211 (69.4%) mothers had a favorable attitude towards exclusive breastfeeding practice. Three hundred one (99%) respondents knew that HIV virus transmits from the mother to their infants and mentioned at least one time when HIV transmits from the mother to the baby. All respondents mentioned at least one way of reducing MTCT of HIV including EBF and others. Two hundred fifty-four (83.6%) knew breast milk alone is sufficient for the first six months of life. But one-six of respondents 50 (16.4%) didn’t know as breast milk alone is sufficient and for how long breast milk alone is given. 118 (38.8%) said they will give expressed breast milk when they had to leave home for long hours for their infants of less than 6 months and 117 (38.5%) and 69 (22.4%) said they will give prepared food and don’t know what to do respectively (data is not shown in tables).

### Exclusive breastfeeding practice

Two hundred ninety-one (95.7%) had breastfed their infants. 280 (92.1%) had initiated breastfeeding within one hour of birth and 12 (3.9%) after one hour. No one reported practicing pre-lacteal feeding. Regarding exclusive breastfeeding, 270 (88.8%) of respondents breastfed exclusively for the first 6 months of life. Twenty (6.6%) and 14 (4.6%) mothers practiced mixed feeding and exclusive replacement feeding respectively (Table 3).

**Table 3:** Percentage of exclusive breastfeeding practice and other feeding practices of HIV positive women visiting public hospitals of Tigray region from July to October, 2016 (N = 304).

**Infant feeding practice**	Frequency	Percentage
Exclusive breastfeeding	270	88.8
Mixed feeding	20	6.6
Exclusive replacement feeding	14	4.6
Total	304	100.0

The most common reason cited for practicing mixed feeding was an illness of the child (65%) which was followed by the illness of the mother (20%). Fear of HIV transmission to their infants (85.7%) was the most common reason cited for practicing exclusive replacement feeding (data is not shown in tables).

### Determinants of exclusive breastfeeding practice

Mothers who were counseled on infant feeding options during ANC of last pregnancy had odds 6.9 times higher to practice exclusive breastfeeding than those who were not counseled [AOR = 6.9, 95% CI; 2.62, 17.99].

Mothers who had favorable attitude had odds 7.9 times higher to practice exclusive breastfeeding than those who had unfavorable attitude [AOR = 7.92, 95% CI; 2.96, 21.21].

Women who had good knowledge on EBF had odds 5.5 times higher [AOR = 5.5, 95% CI; 2.12, 14] to practice exclusive breastfeeding than those who had knowledgeable [AOR = 5.45, 95% CI; 2.12, 14] (Table 4).

**Table 4:** Determinants of exclusive breastfeeding practice of HIV positive women visiting public hospitals, Tigray, Northern Ethiopia, 2016 (N = 304).

**Variables**	Category	Exclusively breastfed		COR (95% Cl)	AOR (95% Cl)
		Yes Freq. (%)	No Freq. (%)		
Educational status	No formal education	70 (83.3)	14 (16.7)	1	1
	Primary education	80 (96.4)	3 (3.6)	5.33(1.472, 19.327)*	2.546 (0.516, 12.568)
	Secondary education	103 (89.6)	12 (10.4)	1.72(0.750, 3.932)*	0.610 (0.176, 2.121)
	More than secondary education	20 (90.9)	2 (9.1)	2.0(0.419, 9.543)	1.407 (0.195, 10.163)
Marital status	Married	226 (90.4)	24 (9.6)	4.71(0.412, 53.860)*	3.237 (0.139, 75.523)
	Single	24 (88.9)	3 (11.1)	4.0(0.273, 58.562)	2.381 (0.076, 4.180)
	Divorced	21 (87.5)	3 (12.5)	3.5(0.238, 51.400)	3.321 (0.107, 103.393)
	Widowed	2 (66.7)	1 (33.3)	1	1
Monthly income	<22.75 USD	16 (84.2)	3 (15.8)	1	1
	22.78–45.5 USD	95 (86.4)	15 (13.6)	1.19(0.308, 4.572)	0.322 (0.051, 2.044)
	> = 45.55 USD	162 (92.6)	13 (7.4)	2.34(0.602, 9.072)*	0.616 (0.085, 4.453)
Occupation	Government employee	54 (94.7)	3 (5.3)	1.20(0.190, 7.586)	0.940 (0.083, 10.589)
	Merchant	50 (92.6)	4 (7.4)	0.83(0.144, 4.828)	0.344 (0.036, 3.339)
	Farmer	11 (73.3)	4 (26.7)	0.18(0.029, 1.146)*	1.563 (0.015, 165.081)
	House wife	128 (87.7)	18 (12.3)	0.48(0.104, 2.155)	0.510 (0.066, 3.928)
	Daily laborer	30 (93.8)	2 (6.2)	1	1
Place of residence	Urban	255 (90.7)	26 (9.3)	2.72(0.935, 7.941)*	1.540 (0.176, 13.462)
	Rural	18 (78.3)	5 (21.7)	1	1
Number of children	One	56 (88.9)	7 (11.1)	2.29(0.394, 13.245)	3.837 (0.524, 28.100)
	Two	101 (86.3)	16 (13.7)	1.80(0.344, 9.463)	1.432 (0.231, 8.867)
	Three to five	109 (94.8)	6 (5.2)	5.19(0.881, 30.572)*	5.132(0.701, 37.579)
	Six and above	7 (77.8)	2 (22.2)	1	1
Disclosure of HIV status	Yes	260 (92.5)	21 (7.5)	9.52(3.733, 24.300)*	2.837 (0.655, 12.290)
	No	13 (56.5)	10 (43.5)	1	1
ANC	Yes	258 (92.5)	21 (7.5)	8.19(3.279, 20.459)*	1.094 (0.193, 6.189)
	No	15 (60)	10 (40.0)	1	1
**Infant feeding counseling during ANC**	Yes	241 (93.8)	16 (6.2)	6.59(2.942, 14.762)*	6.87(2.625, 4.017)**
	No	32 (69.6)	14 (30.4)	1	1
**Attitude towards EBF**	Favorable	202 (95.7)	9 (4.3)	6.96(3.059, 15.811)*	7.92(2.962, 21.212)**
	Unfavorable	71 (76.3)	22 (23.7)	1	1
**Knowledge on EBF**	Good knowledge	244 (95.7)	16 (4.3)	7.89(3.535, 17.603)*	5.45(2.123, 14.017)**
	Poor knowledge	29 (76.3)	15 (23.7)	1	1
Mode of delivery	SVD	243 (90.7)	25 (9.3)	1	1
	Instrumental delivery	23 (82.1)	5 (17.9)	0.47(0.165, 1.354)*	0.556 (0.101, 3.059)
	Caesarian section	7 (87.5)	1 (12.5)	0.72(0.085, 6.099)	0.217 (0.020, 2.387)

* = P-Value ≤0.*20*,

** = P-Value ≤0.*05*

## Discussion

This study assessed magnitude of exclusive breastfeeding and its associated factors among HIV positive women visiting public hospitals of Tigray region. The study design and sampling technique employed in the present study was scientific. Furthermore, the validity and reliability of the instrument was checked intensively and appropriate statistical analysis methods were used. Therefore, the information obtained from the present study is reliable.

In this study, two hundred seventy (88.8%) women practiced EBF for the first six months of age. This finding is much higher than studies carried out in southern Ethiopia (48.2%)[10], west Oromia (72%)[9], Gonder town (83.9%)[14], and Addis Ababa (68%)[15]. The difference might be due difference in culture of feeding habit, study time as well as the availability of resources to practice exclusive breastfeeding.

This study also revealed that infant feeding counseling during ANC, attitude towards EBF and knowledge on EBF were factors associated with EBF practice of HIV positive women.

The observed association between mothers who were counseled on infant feeding options during ANC of last pregnancy and EBF practice of HIV positive women is consistent with another study in Ethiopia and Nigeria[10, 16–18].This implies that providing information on infant feeding options help mothers to choose feeding options in the context of HIV.

Another factor which is associated with EBF practice of HIV positive women is knowledge of mothers, which is supported by several studies[10,18], though another study indicates PMTCT knowledge was not significantly associated with EBF practice[19]. This might be because mothers who have insight on exclusive breastfeeding have a tendency to practice it. This situation calls for a need to intensify education on exclusive breastfeeding for the first six months of life among community members particularly the mothers themselves.

Attitude towards exclusive breastfeeding is also significantly associated with exclusive breastfeeding practice, which is consistent with studies in Ethiopia[11, 18].

In this study, two hundred eleven (69.4%) had a favorable attitude towards EBF practice. The finding of this study is slightly lower than the study done in Mekelle city, which is 81%[11]. The finding of this study is higher than the study done in southern Ethiopia which is 56.7%[10]. This discrepancy might be due to the difference in characteristics of study population and the study area.

The limitations of the present study include; the result of the study might not be generalized to the entire population since this study was conducted in health institutions. Besides, this study employed only quantitative approach, it would have been better if it included qualitative approach to investigate factors in detail. Nevertheless, the study has also several strengths including employing a validated and standardized questionnaire that was tested and revised.

## Conclusions

This study revealed that high proportion of mothers practiced exclusive breastfeeding for the first six months of life.

This study also identified several factors associated with EBF practice of HIV positive women. Infant feeding counseling during ANC, Attitude towards EBF, and Knowledge on EBF were all factors associated with EBF practice of HIV positive women. The Government of Ethiopia must continue to formulate a multidimensional behavioral change strategy to improve mothers’ attitude towards exclusive breastfeeding through involving mothers, family members, and the community at large. Moreover, strengthening information provided regarding infant feeding options during antenatal care and increasing mothers’ knowledge on EBF shall be done to boost EBF practice.

### Ethics approval and consent to participate

The study protocol was approved by the Institutional Research Review Board of Mekelle University’s College of Health Sciences and Community Services Ethical Review Committee (reference number ERC 07878/2016). Permission was obtained from all relevant authorities in the Tigray Regional Health Bureau and hospitals. Informed written consent was obtained from participants prior to enrollment in the study. For participants who were unable to write, a right thumbprint was taken as a signature. Parental or legal guardian consent was obtained for respondents who were under 18 years of age. Participation in the study was voluntary and participants were informed of the right to withdraw from the study. Data collection was conducted confidentially and data de-identified and de-linked and stored in a secure location.

## Supporting information

S1 File (XLSX)

## Abbreviations

EBF: Exclusive breastfeeding
UNICEF: United Nations Children’s Emergency Fund
HIV: Human Immunodeficiency Virus
EDHS: Ethiopian Demographic and Health Survey
ANC: Antenatal Clinic
PMTCT: Prevention of Mother to Child Transmission of HIV
ART: Antiretroviral Therapy
MTCT: Mother-to-child transmission of HIV
SPSS: Statistical Package for Social Science
SVD: Spontaneous Vaginal Delivery
HEI: Human Immunodeficiency Virus exposed infants
SVD: Spontaneous vaginal delivery
